# Integrated stress response restricts macrophage necroptosis

**DOI:** 10.26508/lsa.202101260

**Published:** 2021-11-11

**Authors:** David E Place, Parimal Samir, RK Subbarao Malireddi, Thirumala-Devi Kanneganti

**Affiliations:** Department of Immunology, St. Jude Children’s Research Hospital, Memphis, TN, USA

## Abstract

The integrated stress response (ISR) regulates cellular homeostasis and cell survival following exposure to stressors. Cell death processes such as apoptosis and pyroptosis are known to be modulated by stress responses, but the role of the ISR in necroptosis is poorly understood. Necroptosis is an inflammatory, lytic form of cell death driven by the RIPK3-MLKL signaling axis. Here, we show that macrophages that have induced the ISR are protected from subsequent necroptosis. Consistent with a reduction in necroptosis, phosphorylation of RIPK1, RIPK3, and MLKL is reduced in macrophages pre-treated with ISR-inducing agents that are challenged with necroptosis-inducing triggers. The stress granule component DDX3X, which is involved in ISR-mediated regulation of pyroptosis, is not required for protecting ISR-treated cells from necroptosis. Disruption of stress granule assembly or knockdown of *Perk* restored necroptosis in pre-stressed cells. Together, these findings identify a critical role for the ISR in limiting necroptosis in macrophages.

## Abstract (precis)

Stress inhibits necroptosis in a PERK-dependent manner via reduced RIPK1-RIPK3-MLKL signaling, showing an integral mechanistic connection between stress responses and programmed cell death.

## Introduction

Given the broad range of potential stressors in the environment, cells must carefully balance stress-induced cell death with responding to and recovering from stressors. Programmed cell death pathways are activated by cellular stressors including host-derived inflammatory cytokines, microbial agents, and many other exogenous stresses (Fuchs & Steller, 2011; Galluzzi et al, 2018; Place & Kanneganti, 2019; Tang et al, 2019; Samir et al, 2020; Kesavardhana et al, 2020a; Place et al, 2021). Whereas programmed cell death is critical for organismal development and control of microbial infection, dysregulated and excessive cell death can contribute to autoinflammatory disease, developmental defects, and cancer (Fuchs & Steller, 2011; Place & Kanneganti, 2019; Kesavardhana et al, 2020a; Place et al, 2021). Inhibitory regulation of programmed cell death is therefore critical for limiting cell death-induced pathology. One pathway involved in such regulation, the integrated stress response (ISR), has been shown to inhibit two central programmed cell death pathways, apoptosis and pyroptosis (Arimoto et al, 2008; Thedieck et al, 2013; Samir et al, 2019), but its role in necroptosis is unknown.

Necroptosis is a form of lytic programmed cell death which is initiated in cells with defective apoptosis signaling. In cells where caspase-8 activity is inhibited, inflammatory signaling through TNFR1 results in interaction between RIPK1 and RIPK3 via RIP homotypic-interaction motif (RHIM) domains, phosphorylation of RIPK3, and RIPK3-dependent phosphorylation of MLKL. MLKL oligomerization mediates cell death through disruption of plasma membrane integrity (Sun et al, 2012; Zhao et al, 2012; Wang et al, 2014). Although necroptosis-deficient mice (*Ripk3*^−/−^ or *Mlkl*^−/−^) appear developmentally normal, necroptosis can be activated under various disease states. Physiologically, necroptosis mediated by RIPK3 and MLKL promotes embryonic lethality in *Casp8*^−/−^ mice (Kaiser et al, 2011; Oberst et al, 2011; Alvarez-Diaz et al, 2016). Similarly, RIPK1 RHIM domain mutant mice exhibit perinatal lethality driven by spontaneous, ZBP1-dependent activation of RIPK3 and MLKL (Newton et al, 2016; Kesavardhana et al, 2020a). Necroptosis also drives dermatitis in an epithelial cell-specific RIPK1 knockout mouse model (Dannappel et al, 2014). Recently, a gain-of-function mutation in MLKL, *Mlkl*^D139V^, was found to result in lethal postnatal inflammation in mice, and similar mutations in the human MLKL brace region are associated with chronic recurrent multifocal osteomyelitis, demonstrating MLKL-driven inflammation is a key inflammatory cell death regulator (Hildebrand et al, 2020). MLKL is also important for mediating pathogen clearance in mice (Kitur et al, 2016; Yu et al, 2018; Zhang et al, 2020). In addition to causing necroptosis, MLKL-dependent plasma membrane disruption can also result in activation of NLRP3-dependent pyroptosis, suggesting necroptosis may be important in promoting inflammation in NLRP3 inflammasome-mediated diseases (Conos et al, 2017). Because spontaneous or excessive necroptosis results in significant autoinflammation, we hypothesized that the ISR, which is important for modulating cell fate decisions in response to multiple exogenous cell stressors (Arimoto et al, 2008; Thedieck et al, 2013; Samir et al, 2019), may act as a cell-intrinsic negative regulator of necroptosis.

Mechanistically, the ISR responds to a range of cellular stressors by modulating the activity of the translational machinery while also initiating gene expression required for resolving stress-induced changes to normal cellular homeostasis (Costa-Mattioli & Walter, 2020; Riggs et al, 2020). The ISR is initiated by activation of ISR sensor kinases (including PERK, HRI, GCN2, and PKR) that phosphorylate eIF2α, resulting in sequestration of translational machinery in liquid organelles called stress granules (SGs) made up of proteins including the core component G3BP1 (Wek et al, 1995; Tourrière et al, 2003; McEwen et al, 2005; Taniuchi et al, 2016). Under stress conditions, SG formation can also sequester apoptosis-inducing proteins and limit apoptosis (Arimoto et al, 2008; Thedieck et al, 2013). Recently, recruitment of DDX3X into SGs was also shown to inhibit NLRP3-dependent pyroptosis by sequestration of DDX3X molecules, which are required for NLRP3 inflammasome activation in unstressed cells (Samir et al, 2019). Although sometimes cell protective, dysregulated SG assembly can also promote cell death (Reineke & Neilson, 2019). SG dysfunction is thought to contribute to disease progression in cancer and neurodegeneration (Anderson et al, 2015; Wolozin & Ivanov, 2019). Although SGs have been shown to reduce cell death via apoptosis and pyroptosis (Arimoto et al, 2008; Thedieck et al, 2013; Samir et al, 2019), their influence on necroptosis remains unknown. Many previous studies on the ISR and SGs have used immortalized cell lines which are inherently dysfunctional in normal cell death pathways, notably necroptosis (Su et al, 2016). We therefore examined the potential role for the ISR in regulating necroptosis in primary macrophages in this study.

Here, we identify a critical role for the ISR in protecting macrophages from necroptotic cell death. Macrophages pretreated with stress-inducing agents were resistant to subsequent death when challenged with necroptosis triggers. Mechanistically, pre-stressed macrophages treated with necroptosis triggers failed to activate the key necroptosis signaling proteins RIPK1, RIPK3, and MLKL. Stress-mediated protection from necroptosis did not require the SG component DDX3X, unlike stress-mediated protection from NLRP3-dependent pyroptosis. Disruption of SG assembly or knockdown of PERK-dependent ISR signaling restored necroptosis in stressed cells. Together, these findings suggest a critical role for the ISR in regulating necroptosis.

## Results

### SG triggers inhibit macrophage necroptosis

**Figure 1.**
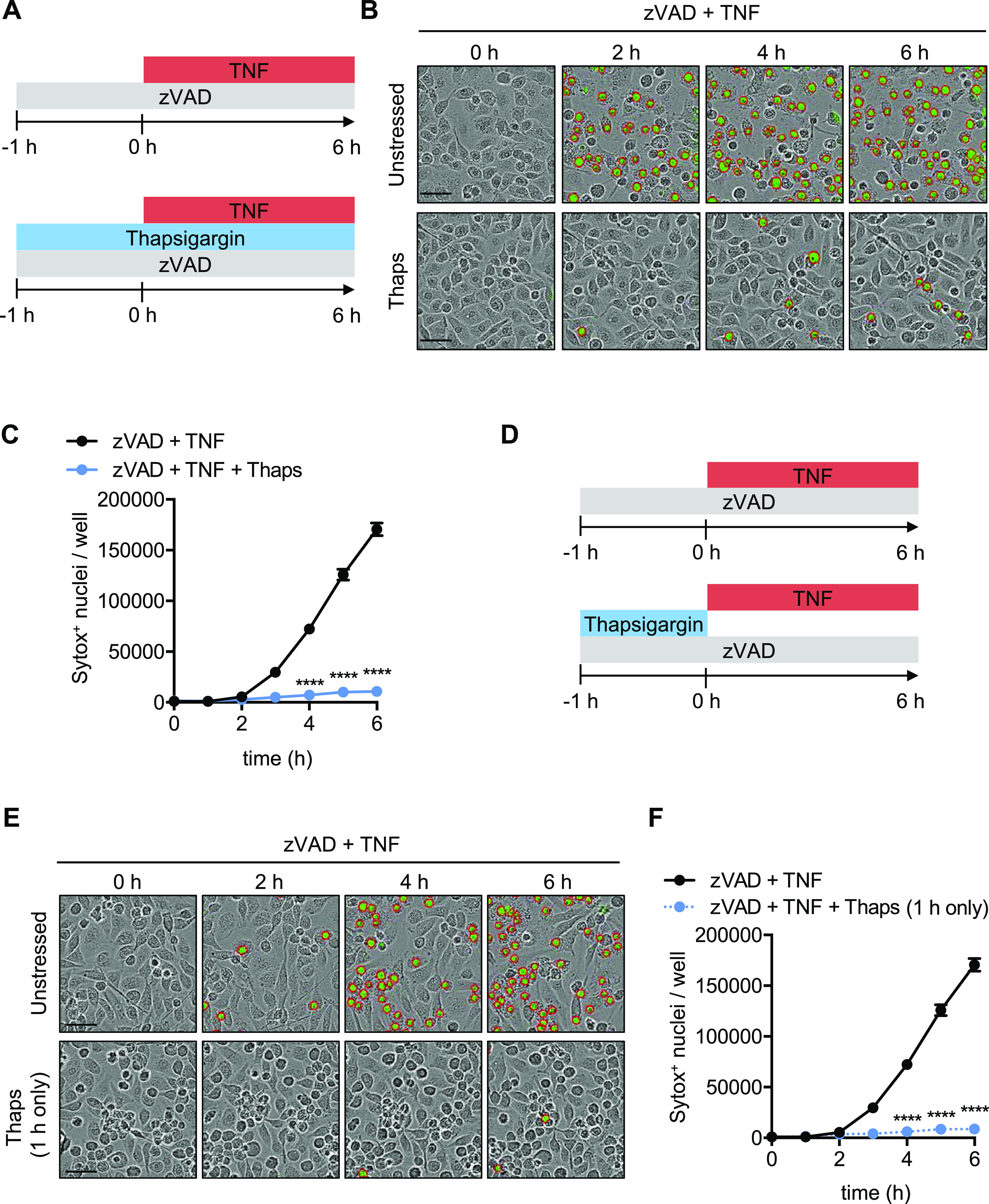
Stress granule triggers inhibit macrophage necroptosis. Primary BMDMs were stimulated as indicated. **(A)** Schematic for inducing necroptosis (zVAD + TNF) in unstressed or pre-stressed (thapsigargin [Thaps] treated) BMDMs. **(B)** Representative IncuCyte images collected at indicated time-points after addition of TNF, where necroptotic cells were quantified by uptake of membrane-impermeant Sytox Green (green, with red analysis mask outline). **(C)** Quantification of necroptosis from automated image analysis of Sytox Green–positive nuclei at indicated time-points. **(D)** Schematic for acute pre-stressing of BMDMs with 1 h thapsigargin treatment before necroptosis induction with TNF. **(E)** Representative IncuCyte images of necroptosis quantification in acute thapsigargin pre-stressed BMDMs (green, with red analysis mask outline). **(F)** Quantification of necroptosis from automated analysis for acute thapsigargin pre-stressed BMDMs. Significance was determined (C, F) by two-way ANOVA followed by Dunnett’s multiple comparisons test (versus zVAD + TNF), ****P < 0.0001. Data are generated from three images per replicate well (n = 3) and are representative of at least three independent biological replicate experiments. Scale bar (black) indicates 50 μm. Data are presented as mean ± SEM.

**Figure S1.**
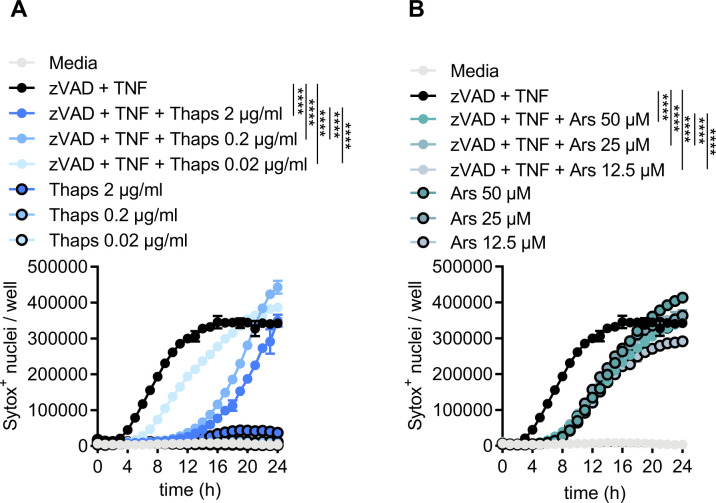
Thapsigargin and arsenite pre-stress dose–response time course. **(A, B)** Primary BMDMs were stimulated as indicated with or without thapsigargin (Thaps) (A) or with or without arsenite (Ars) (B). Quantification of necroptosis from automated image analysis of Sytox Green–positive nuclei at indicated time-points. Significance was determined by two-way ANOVA followed by Tukey’s multiple comparisons test, ****P < 0.0001. Data are generated from three images per replicate well (n = 3) and are representative of at least two independent biological replicate experiments. Data are presented as mean ± SEM.

**Figure S2.**
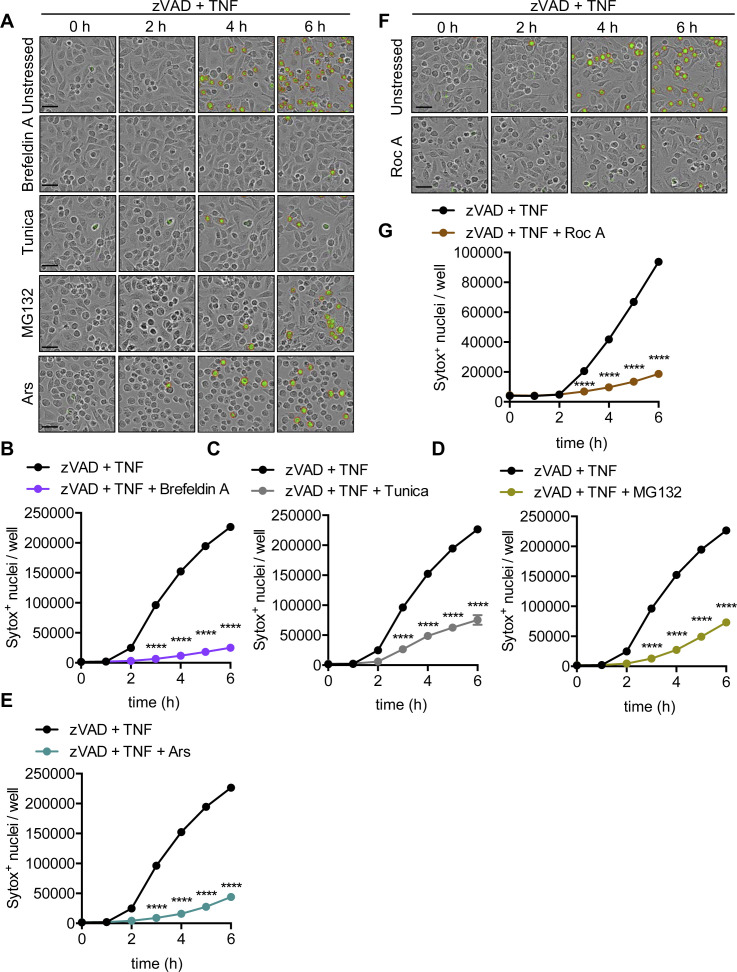
Multiple stress granule inducers protect BMDMs from TNF-mediated necroptosis. Primary BMDMs were stimulated with zVAD + TNF and pre-stressed as indicated. **(A, F)** Representative IncuCyte images collected at indicated time-points following addition of TNF, where necroptotic cells were quantified by uptake of membrane-impermeant Sytox Green (green, with red analysis mask outline). **(B, C, D, E, G)** Quantification of necroptosis from automated image analysis of Sytox Green–positive nuclei at indicated time-points in cells pre-stressed with (B) brefeldin A, (C) tunicamycin (Tunica), (D) MG132, (E) arsenite (Ars), or (G) rocaglamide A (Roc A). Significance was determined (B, C, D, E, G) by two-way ANOVA followed by (B, C, D, E) Dunnett’s or (G) Sidak’s multiple comparisons test (versus zVAD + TNF), ****P < 0.0001. Data are generated from three images per replicate well (n = 3) and are representative of at least three independent biological replicate experiments. Scale bar (black) indicates 50 μm. Data are presented as mean ± SEM.

**Figure S3.**
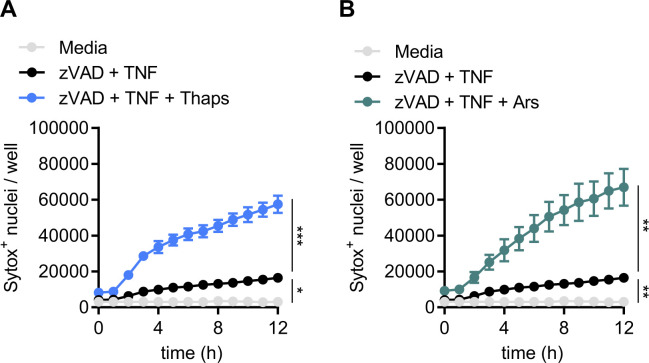
Primary MEF necroptosis is not inhibited by integrated stress response. **(A, B)** Primary MEFs were stimulated with zVAD + TNF and pre-stressed with (A) thapsigargin (Thaps) or (B) arsenite (Ars). Quantification of necroptosis from automated image analysis of Sytox Green–positive nuclei at indicated time-points. Significance was determined by two-way ANOVA followed by Tukey’s multiple comparisons test, *P < 0.05, **P < 0.01, and ***P < 0.001. Data are generated from three images per replicate well (n = 3) and are representative of at least two independent biological replicate experiments. Data are presented as mean ± SEM.

To determine whether the ISR can inhibit necroptosis, we pretreated BMDMs with the SG-inducing agent thapsigargin, which induces ER stress (Fig 1A) (Harding et al, 2000). To trigger TNFR1-dependent necroptosis, caspase-8 activity must be inhibited during TNF treatment; therefore, we also added the pan-caspase inhibitor zVAD to BMDMs before the addition of TNF (Fig 1A). In BMDMs pretreated with thapsigargin, necroptotic cell death was significantly reduced compared to unstressed cells (Figs 1B and C and S1A). To determine whether acute thapsigargin-induced stress also limited necroptosis, we pretreated cells for 1 h and washed off thapsigargin before stimulating BMDMs with the necroptosis trigger zVAD plus TNF (Fig 1D). Consistent with prolonged thapsigargin treatment (Fig 1A–C), acute thapsigargin pretreatment also inhibited cell death after stimulation with necroptosis triggers (Fig 1E and F). To further determine whether the ISR inhibits necroptosis, we pre-stressed BMDMs with additional SG inducers including brefeldin A, tunicamycin, MG132, arsenite, and rocaglamide A. Consistent with the thapsigargin-induced stress-mediated reduction of necroptosis, brefeldin A, tunicamycin, MG132, arsenite, and rocaglamide A pre-stressed BMDMs were protected from TNFR1-dependent necroptosis (Figs S1A and B and S2A–G). MEFs also undergo necroptosis after zVAD and TNF treatment; however, pretreatment with thapsigargin or arsenite enhanced cell death rather than reducing it as in BMDMs, suggesting cell type–specific responses guide the cell fate choice after stress and necroptosis signaling (Fig S3A and B).

**Figure S4.**
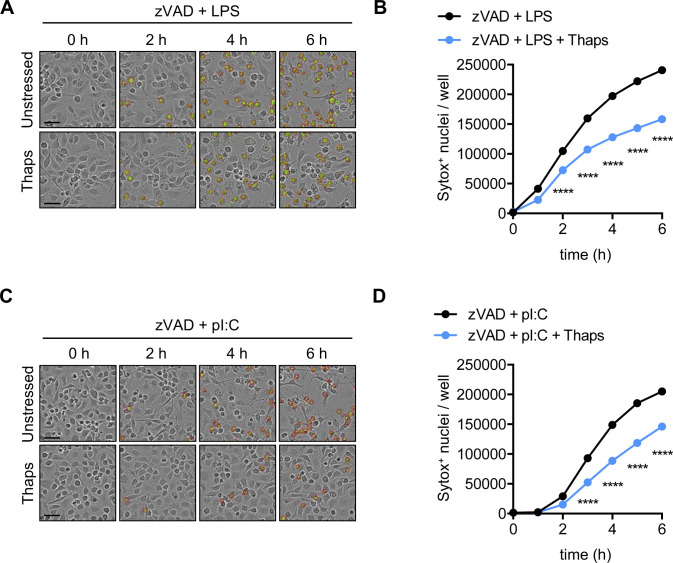
TLR driven necroptosis is inhibited by stress granule triggers. Primary BMDMs were stimulated as indicated and pre-stressed with thapsigargin (Thaps). **(A, C)** Representative IncuCyte images collected at indicated time-points following addition of (A) zVAD + LPS or (C) zVAD + poly I:C (pI:C), where necroptotic cells were quantified by uptake of membrane-impermeant Sytox Green (green, with red analysis mask outline). **(B, D)** Quantification of necroptosis from automated image analysis of Sytox Green–positive nuclei at indicated time-points in cells pre-stressed with thapsigargin and stimulated with (B) zVAD + LPS or (D) zVAD + poly I:C. Significance was determined (B, D) by two-way ANOVA followed by Sidak’s multiple comparisons test, ****P < 0.0001. Data are generated from three images per replicate well (n = 3) and are representative of at least three independent biological replicate experiments. Scale bar (black) indicates 50 μm. Data are presented as mean ± SEM.

**Figure S5.**
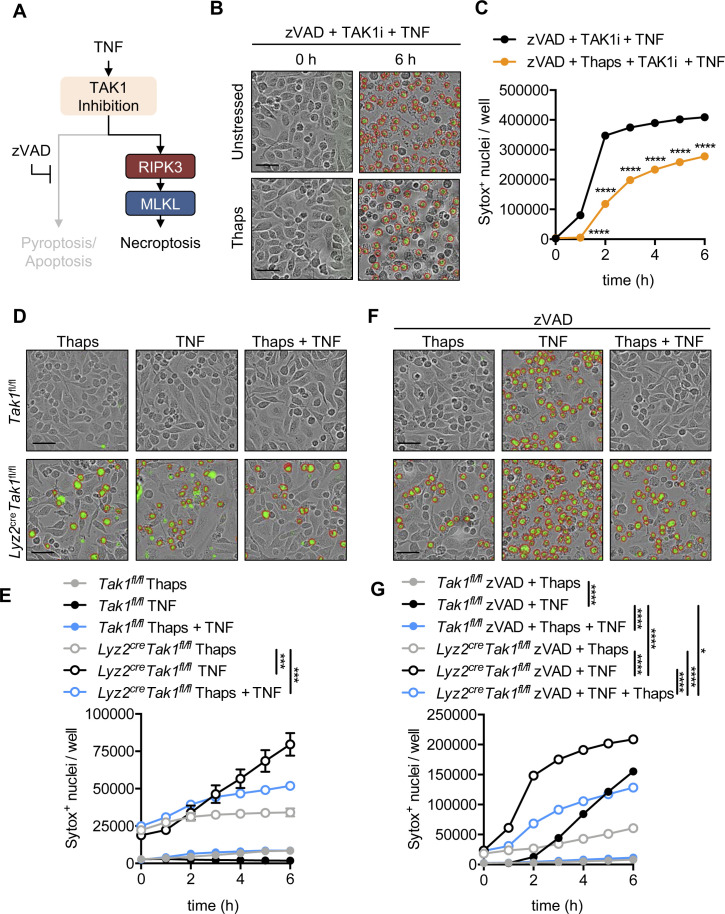
Pre-stressed BMDMs lacking TAK1 activity are protected from cell death. Primary BMDMs were stimulated as indicated. **(A)** Schematic for inducing necroptosis (zVAD + TAK1i + TNF) in unstressed or pre-stressed (thapsigargin [Thaps] treated) wildtype BMDMs. **(B)** Representative IncuCyte images collected at indicated time-points following addition of zVAD + TAK1 inhibitor (TAK1i) + TNF with or without thapsigargin (Thaps) pretreatment, where necroptotic cells were quantified by uptake of membrane-impermeant Sytox Green (green, with red analysis mask outline). **(C)** Quantification of cell death from automated image analysis of Sytox Green–positive nuclei at indicated time-points. **(D, E, F, G)** Primary BMDMs derived from control (*Tak1*^fl/fl^) or myeloid-specific *Lyz2*^cre+^*Tak1*^fl/fl^ mice were treated as indicated, and representative images were obtained from automated IncuCyte analysis where cell death was quantified by uptake of membrane-impermeant Sytox Green (green, with red analysis mask outline). Significance was determined (C, E, G) by two-way ANOVA followed by (C) Sidak’s or (E, G) Tukey’s multiple comparisons test, *P < 0.05, ***P < 0.001, and ****P < 0.0001. Data are generated from three images per replicate well (n = 3) and are representative of at least three independent biological replicate experiments. Scale bar (black) indicates 50 μm. Data are presented as mean ± SEM.

TNF signaling via TNFR1 drives necroptosis through the RIPK1-RIPK3-MLKL signaling axis (Sun et al, 2012; Zhao et al, 2012). Toll-like receptor signaling via the adaptor TRIF (which contains a RHIM domain) engages necroptosis in a RIPK3-dependent manner that does not require RIPK1 (Kaiser et al, 2013; Malireddi et al, 2020a). Similar to the observed impact on TNFR1-mediated necroptosis, LPS signaling via TLR4 (Fig S4A and B) and poly I:C signaling via TLR3 (Fig S4C and D), which both signal through TRIF, induced necroptosis in cells treated with zVAD, but necroptosis was reduced in thapsigargin pre-stressed cells. Inhibition of the MAP kinase TAK1 (also known as MAP3K7) can also induce activation of pyroptotic, apoptotic, and/or necroptotic molecules and cause PANoptosis (Malireddi et al, 2020a). Pretreatment of cells with zVAD followed by TAK1 inhibitor (5Z-7-oxozeaenol; TAK1i) results in RIPK1 kinase function-independent, but RIPK1 scaffolding function-dependent, MLKL-dependent necroptosis (Malireddi et al, 2020a). Similar to necroptosis induced by TNFR1 and TLRs, TAK1 inhibition with concurrent pan-caspase inhibition (zVAD) resulted in cell death which was limited in thapsigargin pretreated cells (Fig S5A–C). Conditional deletion of TAK1 in *Lyz2*^cre^*Tak1*^fl/fl^ BMDMs also results in spontaneous PANoptosis which is potentiated by TNF (Malireddi et al, 2018, 2020a). Addition of thapsigargin before TNF stimulation in TAK1-deficient BMDMs also reduced cell death (Fig S5D and E). Similar to wildtype BMDMs, TAK1-deficient BMDMs stressed with thapsigargin were also protected from zVAD plus TNF-induced necroptosis (Fig S5F and G). Together, these findings show that induction of the ISR in BMDMs protects from subsequent necroptosis via TNFR1 and TLR-mediated signaling pathways.

### SG inducers inhibit RIPK1, RIPK3, and MLKL phosphorylation

**Figure 2.**
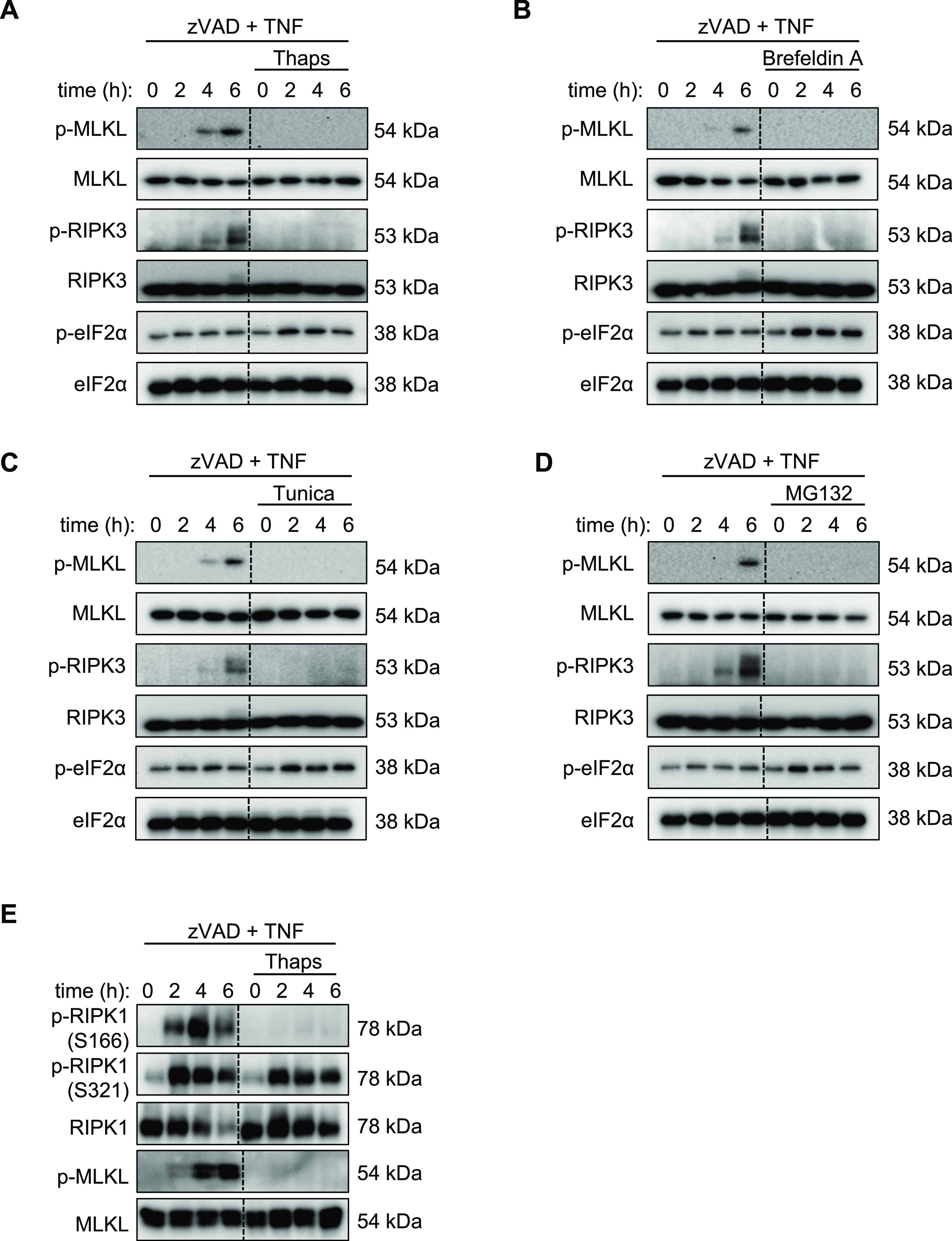
Stress granule inducers inhibit RIPK1, RIPK3, and MLKL phosphorylation. Necroptosis signaling was analyzed by immunoblotting at the indicated time after the induction of necroptosis by TNF treatment. **(A, B, C, D, E)** Lysates from primary BMDMs pre-stressed with (A) thapsigargin (Thaps), (B) brefeldin A, (C) tunicamycin (Tunica), (D) MG132, or (E) Thaps and treated with zVAD + TNF were collected at the indicated time-points. Necroptosis signaling was examined by immunostaining for phosphorylation of MLKL and RIPK3 (p-MLKL and p-RIPK3) and phosphorylation of RIPK1 (p-RIPK1) at serine-166 (S166) or serine-321 (S321) and activation of the integrated stress response by phosphorylation of eIF2α (p-eIF2α). Immunoblots are representative of at least two independent experiments.

Necroptosis is driven by phosphorylation of RIPK3 and MLKL downstream of TNFR1 (Sun et al, 2012; Zhao et al, 2012). To determine whether the ISR inhibits necroptosis via restricting the RIPK3-MLKL signaling axis, we pretreated cells with stress-inducing agents (thapsigargin, brefeldin A, tunicamycin, or MG132) and zVAD (1 h) followed by TNF. Consistent with stress-induced protection from necroptotic cell death (Figs 1 and S2), phosphorylation of MLKL and RIPK3 was reduced in cells pre-stressed with thapsigargin (Fig 2A), brefeldin A (Fig 2B), tunicamycin (Fig 2C), and MG132 (Fig 2D). We also confirmed that each stressor increased phosphorylation of eIF2, an indicator of ISR activation (Fig 2A–D). Upstream of RIPK3, RIPK1 phosphorylation at serine residue 166 is associated with increased necroptosis signaling (Laurien et al, 2020). Consistent with the observed reduction of RIPK3 and MLKL phosphorylation, RIPK1 phosphorylation at S166 but not S321 was reduced in thapsigargin pre-stressed cells (Fig 2E). Together, these data suggest that the ISR limits necroptosis signaling by interfering with RIPK1-RIPK3-MLKL signaling cascade downstream of the TNF receptor.

### DDX3X-dependent SGs are not required for inhibition of necroptosis

**Figure S6.**
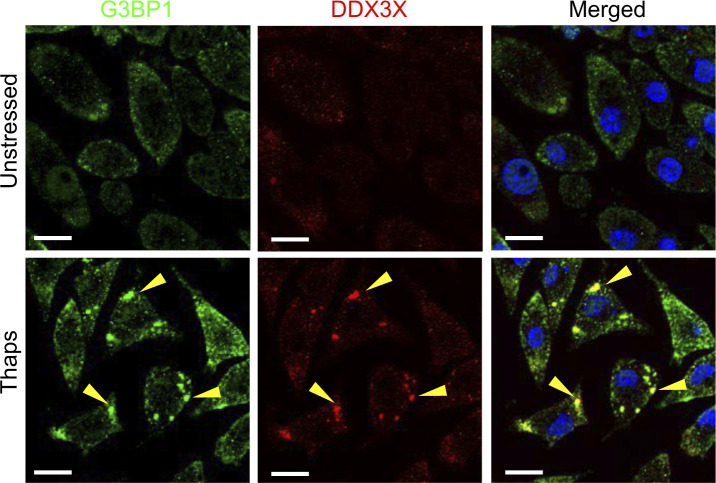
BMDMs form stress granules (SGs) in response to thapsigargin treatment. Primary BMDMs were unstressed or treated with thapsigargin (Thaps) to induce SG formation. Cells were stained for G3BP1 (green), DDX3X (red), and DAPI (blue) to examine SG formation by confocal microscopy. Scale bars (white) indicate 20 μm. Images are representative from at least two independent experiments.

**Figure 3.**
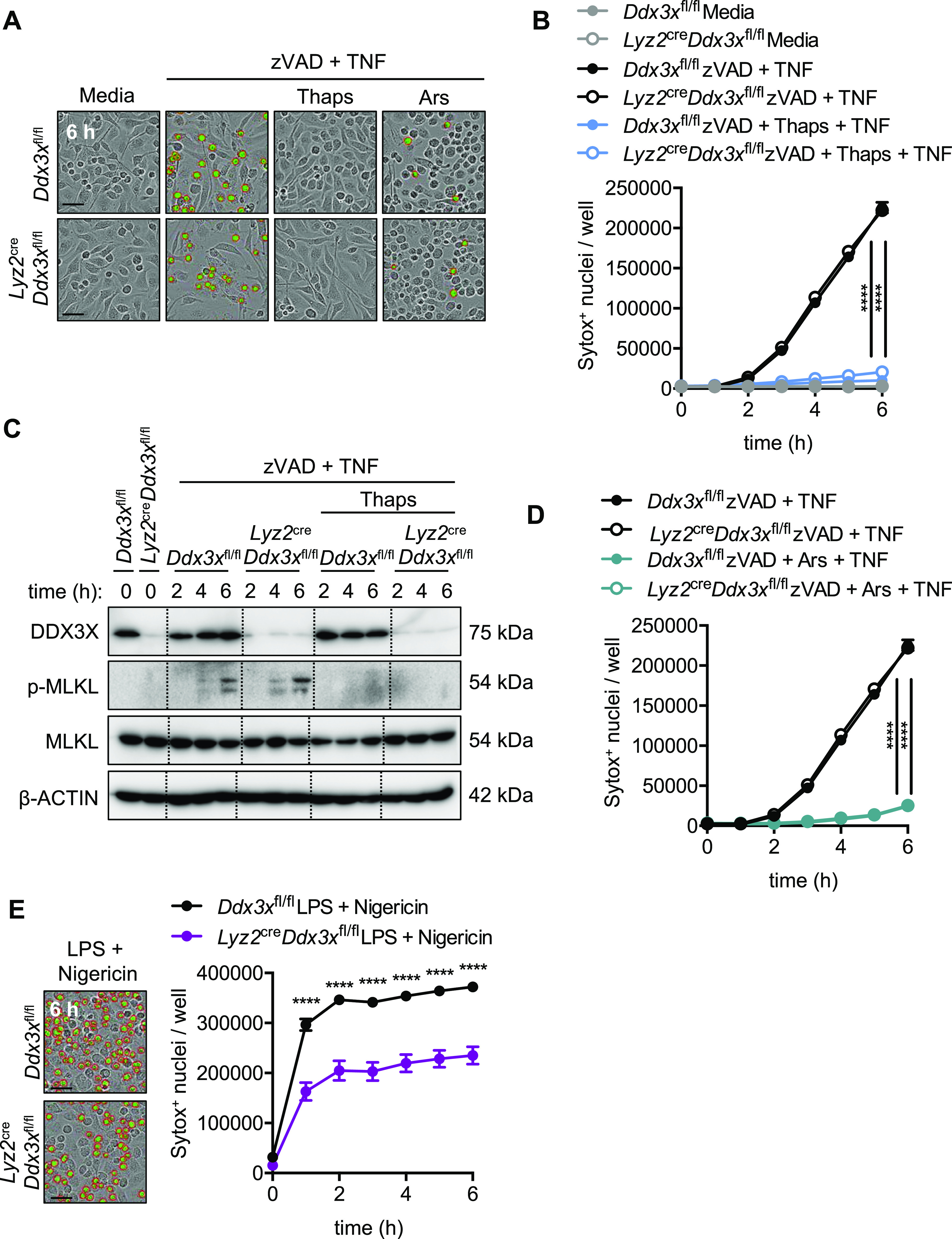
DDX3X is not required for stress-dependent inhibition of necroptosis. Primary BMDMs were stimulated as indicated. **(A)** Primary BMDMs derived from control (*Ddx3x*^fl/fl^) or myeloid-specific *Lyz2*^cre^*Ddx3x*^fl/fl^ mice were treated as indicated, and representative images (6 h) were obtained from automated IncuCyte analysis where necroptotic cells were quantified by uptake of membrane-impermeant Sytox Green (green, with red analysis mask outline). **(B)** Necroptosis was quantified by automated analysis of Sytox Green–positive nuclei. **(C)** Immunoblots were performed from BMDM lysates at the indicated time-points to confirm DDX3X expression was reduced and assess necroptosis signaling (via phosphorylation of MLKL [p-MLKL]) in unstressed or thapsigargin-stressed BMDMs treated with zVAD + TNF. **(D)** Primary BMDMs derived from control (*Ddx3x*^fl/fl^) or myeloid-specific *Lyz2*^cre^*Ddx3x*^fl/fl^ mice were treated as indicated, and necroptosis was quantified by automated analysis of Sytox Green–positive nuclei. *Ddx3x*^fl/fl^ and *Lyz2*^cre^*Ddx3x*^fl/fl^ quantifications overlap upon zVAD + Ars + TNF treatment. **(E)** Representative images (6 h) and IncuCyte quantification of NLRP3-dependent pyroptosis (via LPS + nigericin treatment) in BMDMs derived from the indicated genotypes. Significance was determined (B, D, E) by two-way ANOVA followed by (B, D) Dunnett’s or (E) Sidak’s multiple comparisons test, ****P < 0.0001. Data are generated from three images per replicate well (n = 3) and are representative of at least three independent biological replicate experiments. Scale bar (black) indicates 50 μm. Data are presented as mean ± SEM.

The ISR promotes the condensation of translational machinery and associated proteins into structures termed SGs that are composed of core and accessory proteins. The SG protein DDX3X was recently found to regulate the cell survival and pyroptotic cell fate of BMDMs (Samir et al, 2019). In sodium arsenite pre-stressed cells, DDX3X localizes to SGs; consequently, this sequestration of DDX3X protects BMDMs from NLRP3-dependent pyroptosis. In unstressed cells, DDX3X promotes the activation of NLRP3-dependent pyroptosis (Samir et al, 2019). We therefore examined whether DDX3X was required for ISR-mediated inhibition of necroptosis. First, we confirmed that BMDMs treated with thapsigargin induced the formation of SGs by staining for G3BP1 and DDX3X. Consistent with sodium arsenite-induced stress (Samir et al, 2019), thapsigargin treatment resulted in formation of SGs containing both G3BP1 and DDX3X (Fig S6). To determine whether DDX3X was required for stress-induced protection from necroptosis, we pre-treated control and *Lyz2*^cre^*Ddx3x*^fl/fl^ BMDMs with thapsigargin and zVAD before TNF. BMDMs deficient in DDX3X underwent similar necroptosis to wildtype BMDMs, and thapsigargin stress-induced inhibition of necroptosis did not require DDX3X (Fig 3A and B). Cre-mediated deletion of *Ddx3x* was confirmed by immunoblotting for DDX3X protein in BMDMs (Fig 3C). Consistent with no difference being found in cell death between wildtype and DDX3X-deficient BMDMs, phosphorylation of MLKL was similar in wildtype and DDX3X-deficient BMDMs treated with zVAD plus TNF (Fig 3C). Similar to the thapsigargin-mediated reduction of necroptosis, arsenite similarly restricted cell death in wildtype and DDX3X-deficient BMDMs (Fig 3D). We further confirmed that deletion of DDX3X limits NLRP3-dependent pyroptosis, as previously observed (Samir et al, 2019), by treating control and *Lyz2*^cre^*Ddx3x*^fl/fl^ BMDMs with LPS plus nigericin (Fig 3E). Together, these data suggest that the ISR restricts necroptosis in a DDX3X-independent manner, which is distinct from the role for the ISR and DDX3X in regulating pyroptosis.

### Disruption of SGs restores necroptosis in stressed BMDMs

**Figure 4.**
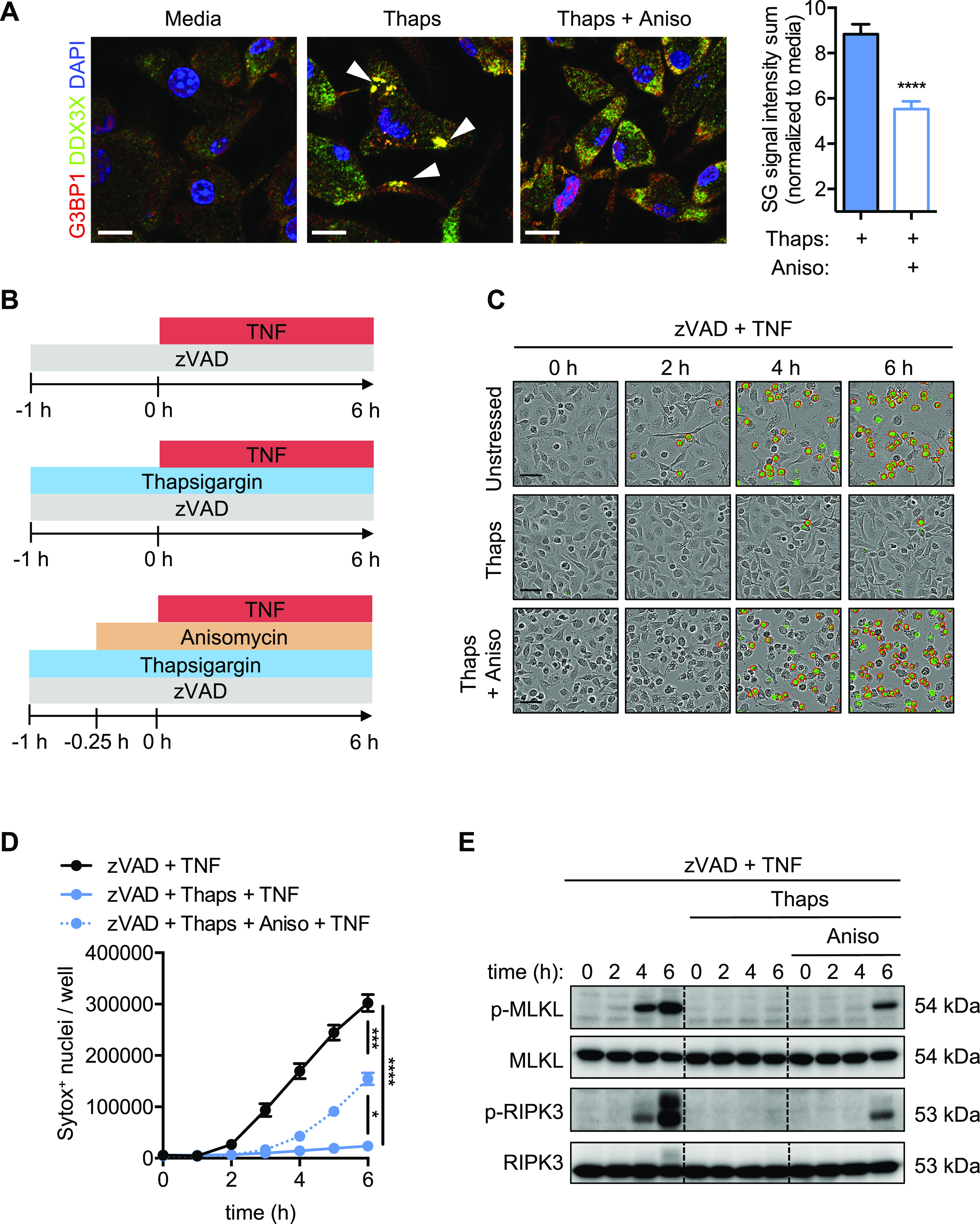
Disruption of SGs restores necroptosis in stressed BMDMs. Disruption of SGs by anisomycin (Aniso) treatment in thapsigargin (Thaps) pre-stressed cells was examined. **(A)** Confocal microscopy images were obtained from primary BMDMs treated as indicated and stained for G3BP1 (red), DDX3X (green), and DAPI (blue), and SG signal intensities were compared. **(B)** Schematic for disrupting SGs in thapsigargin pre-stressed cells treated with Aniso before necroptosis induction. **(C, D)** Representative images of BMDMs (treated as indicated in panel B) and quantification of necroptosis (D) by uptake of membrane-impermeant Sytox Green (green, with red analysis mask outline). **(E)** Immunoblots were performed to determine the effect of SG disruption by Aniso on necroptosis signaling. Significance was determined (A) by Mann–Whitney test or (D) by two-way ANOVA followed by Tukey’s multiple comparisons test, *P < 0.05, ***P < 0.001, and ****P < 0.0001. Data are representative of (A) at least three images per condition or (C, D) from three images per replicate well (n = 3) and are representative of at least three independent biological replicate experiments. Scale bars (black) indicate 50 μm or (white) 20 μm. Data are presented as mean ± SEM.

**Figure S7.**
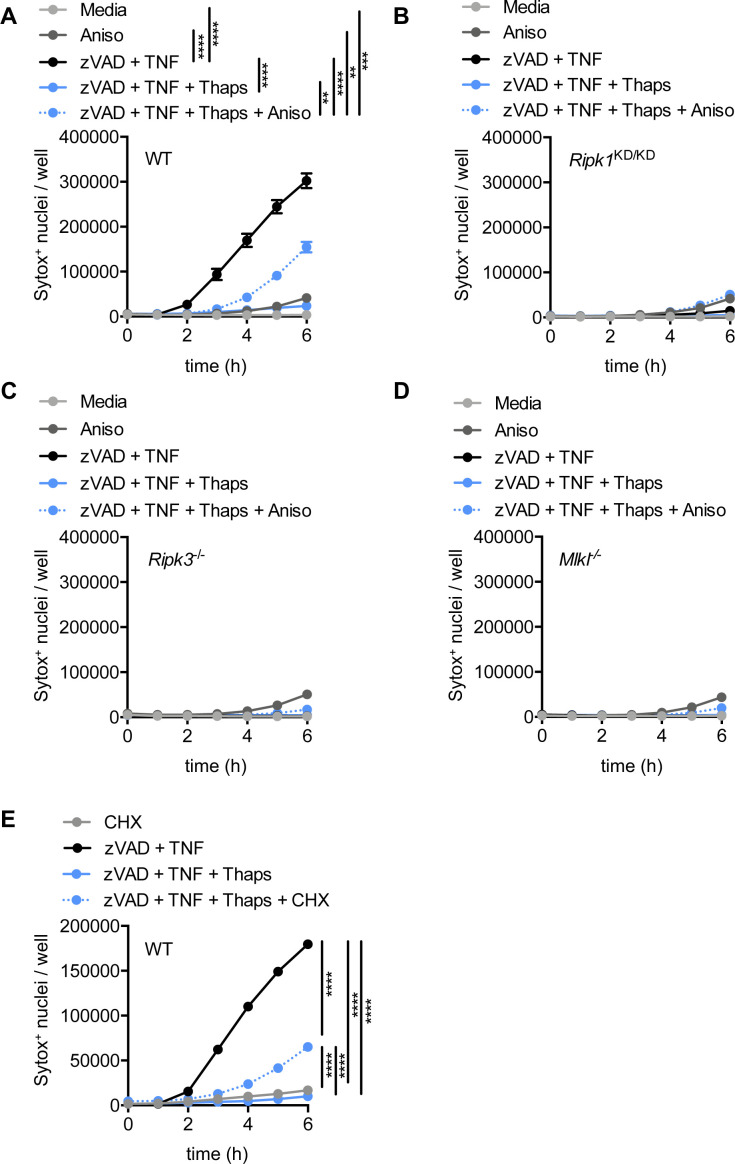
Inhibitors of translation elongation restore necroptosis in thapsigargin-stressed BMDMs. Primary BMDMs were stimulated as indicated. **(A, B, C, D)** Quantification of cell death from automated image analysis of Sytox Green–positive nuclei at indicated time-points in (A) WT BMDMs, (B) *Ripk1*^KD/KD^ BMDMs, (C) *Ripk3*^−/−^ BMDMs, or (D) *Mlkl*^−/−^ BMDMs. **(E)** WT BMDM cell death was similarly quantified at indicated time-points in pre-stressed (thapsigargin [Thaps]-treated) cells subsequently treated with cycloheximide (CHX). Significance was determined by two-way ANOVA followed by Tukey’s multiple comparisons test, **P < 0.01, ***P < 0.001, and ****P < 0.0001. Data are generated from three images per replicate well (n = 3) and are representative of at least three independent biological replicate experiments. Data are presented as mean ± SEM.

SG assembly is a dynamic process driven by inhibition of translation initiation, which leads to the accumulation of stalled pre-initiation complexes and liquid–liquid phase separation of RNA and proteins (Kedersha et al, 1999). To experimentally determine whether disruption of SGs restores necroptosis in BMDMs, we treated cells with the translational elongation inhibitor anisomycin. Anisomycin and other inhibitors of translation elongation stabilize polysomes, inhibit SG assembly, and disrupt pre-formed SGs (Kedersha et al, 2000; Wheeler et al, 2016; Samir et al, 2019). First, we confirmed that anisomycin treatment disrupted pre-formed SGs in thapsigargin-stressed BMDMs. As expected, anisomycin treatment (15 min) led to a rapid reduction of SGs (Fig 4A). We then compared necroptotic cell death in BMDMs that were pre-stressed, stressed and treated with anisomycin to disrupt SGs, or treated with necroptosis-inducing agents alone (Fig 4B). Disruption of SGs with anisomycin restored cell death and phosphorylation of MLKL and RIPK3 in BMDMs treated with zVAD/TNF (Fig 4C–E). In addition, we confirmed that necroptosis was restored by similarly treating wildtype, *Ripk1*^KD/KD^ (K45A/K45A mutation; kinase-dead), *Ripk3*^−/−^, and *Mlkl*^−/−^ BMDMs; we observed cell death in wildtype BMDMs treated with TNF and zVAD that was blocked by thapsigargin treatment and restored in response to anisomycin, whereas there was no induction of cell death in necroptosis-deficient BMDMs (Fig S7A–D). We also confirmed that cycloheximide, another translation elongation inhibitor that disrupts SGs, restored necroptosis (Fig S7E). Together, these data suggest that SG assembly limits necroptotic signaling and cell death.

### PERK-dependent signaling is required for stress-mediated inhibition of necroptosis

**Figure 5.**
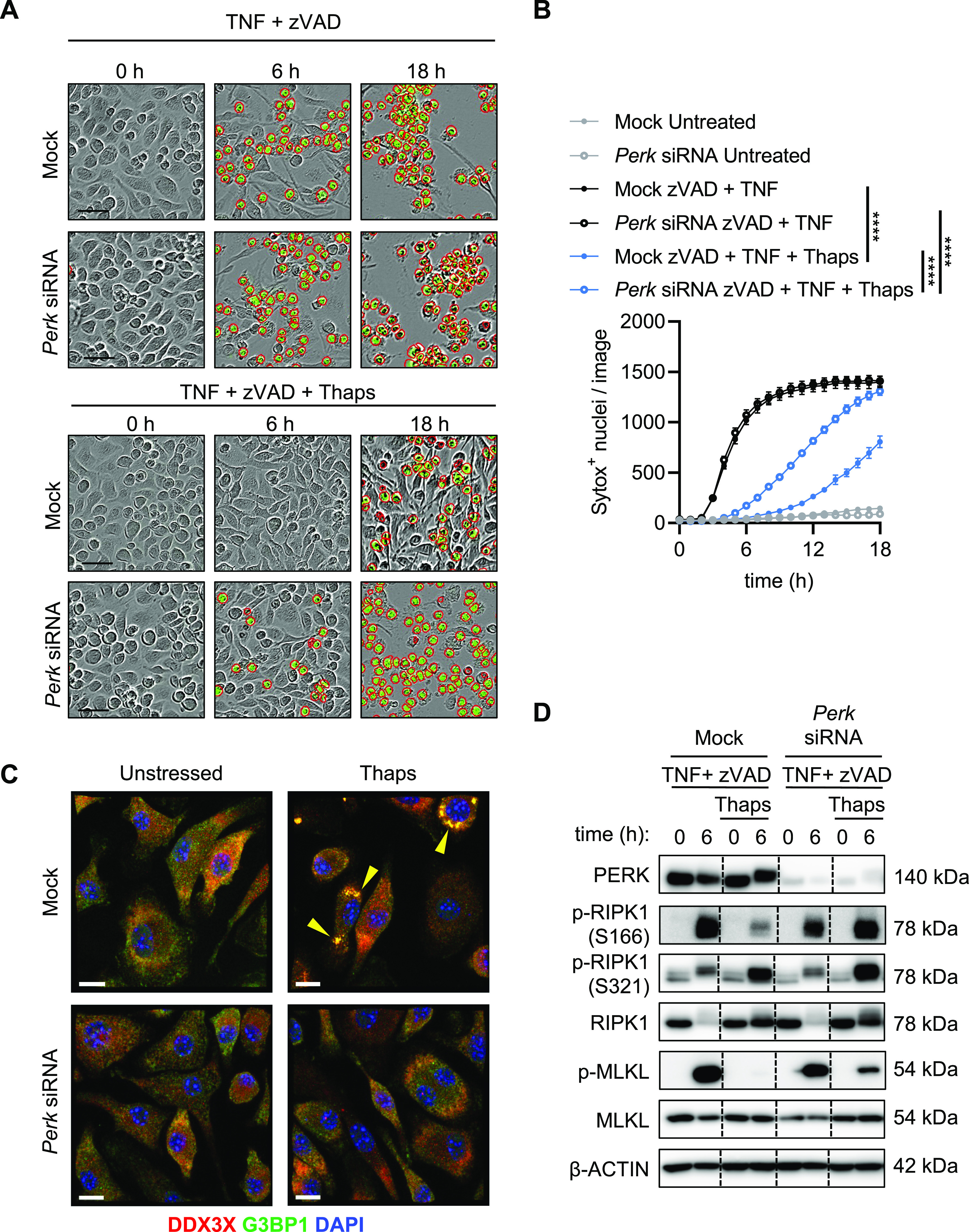
Knockdown of *Perk* restores necroptosis in thapsigargin pre-stressed BMDMs. Primary BMDMs were stimulated as indicated. **(A, B)** Representative IncuCyte images of mock siRNA or *Perk* siRNA knockdown BMDMs treated as indicated (A) and necroptosis was quantified (B) by uptake of membrane-impermeant Sytox Green (green, with red analysis mask outline). **(C)** Confocal images of mock siRNA or *Perk* siRNA treated BMDMs treated as indicated and stained for G3BP1 (green), DDX3X (red), and DAPI (blue). **(D)** Immunoblots were performed to determine whether siRNA (mock or *Perk*) knockdown restored necroptosis signaling in pre-stressed BMDMs. Data are representative of a single independent biological replicate experiment with IncuCyte quantification performed on two replicate wells containing a total of eight image fields. Significance was determined by two-way ANOVA followed by Tukey’s multiple comparisons test (B), ****P < 0.0001. Data are presented as mean ± SEM. Scale bars indicate (black) 50 μm or (white) 10 μm.

Activation of the ISR by thapsigargin involves signaling via PERK (Harding et al, 2000). To genetically determine whether PERK was required for thapsigargin stress-mediated inhibition of necroptosis in BMDMs, we performed siRNA knockdown of *Perk*. Knockdown of *Perk* did not render BMDMs more sensitive to necroptosis following zVAD and TNF treatment (Fig 5A and B). Knockdown of *Perk* did, however, partially restore necroptosis in BMDMs that were pre-stressed with thapsigargin before necroptosis induction (Fig 5A and B). PERK was also required for SG assembly in BMDMs after thapsigargin treatment (Fig 5C). *Perk* knockdown also restored necroptosis signaling via the RIPK1-RIPK3-MLKL axis in thapsigargin pre-stressed BMDMs (Fig 5D). Together, these data suggest that PERK-dependent signaling after thapsigargin-induced stress limits necroptosis in BMDMs by inhibiting the necroptosis signaling cascade downstream of TNFR1.

## Discussion

In this study, we identified a regulatory pathway whereby stress responses in macrophages protect cells from necroptosis. Multiple stress inducers reduced necroptotic cell death induced by necroptosis triggers that signal through TNFR1, TLRs, or by inhibiting TAK1. Consistent with the stress-induced protection from cell death, necroptosis signaling mediated by phosphorylation of RIPK1, RIPK3, and MLKL was inhibited in stressed cells. Disruption of SG formation with the translation inhibitors anisomycin or cycloheximide, or knockdown of *Perk*, restored necroptosis in pre-stressed BMDMs, suggesting that the ISR and downstream SG assembly mediate the protection from necroptosis in pre-stressed macrophages. Previous studies have found that apoptosis and NLRP3-dependent pyroptosis are modulated by the ISR (Arimoto et al, 2008; Samir et al, 2019), but our findings here are the first to show a clear role for the ISR in negatively regulating necroptosis.

Programmed cell death pathways have emerged as critical regulators of inflammatory responses during infection, autoimmune and autoinflammatory diseases, and in cancers. More recently, the extensive crosstalk between cell death pathways has led to the concept of PANoptosis, which is defined as an inflammatory programmed cell death pathway regulated by the PANoptosome complex with key features of pyroptosis, apoptosis, and/or necroptosis that cannot be accounted for by any of these pathways alone. The PANoptosome provides a molecular scaffold for contemporaneous engagement of key molecules from pyroptosis, apoptosis, and necroptosis (Lamkanfi et al, 2008; Malireddi et al, 2010, 2018, 2019, 2020a, 2020b; Gurung et al, 2014, 2016; Lukens et al, 2014; Kuriakose et al, 2016; Banoth et al, 2020; Christgen et al, 2020; Karki et al, 2020a, 2020b, 2021; Kesavardhana et al, 2020b; Lee et al, 2021). Given the inflammatory consequences of cell death, cells possess multiple negative regulatory pathways that restrict excessive inflammation. The ISR and SGs are emerging as an important signaling network responsible for maintaining cellular homeostasis. Recently, the SG-associated protein DDX3X was shown to promote NLRP3 inflammasome activation in unstressed macrophages. In stressed macrophages, DDX3X is sequestered in SGs, limiting activation of the NLRP3 inflammasome (Samir et al, 2019). In our study, however, DDX3X did not appear to play a role in ISR-mediated inhibition of necroptosis. These findings suggest different SG components are involved in regulating pyroptosis and necroptosis. The specific mechanisms by which the ISR inhibits necroptosis are unclear but likely involve signaling components downstream of the TNFR1 involved in necroptosis signaling. Previously, TRAF2, an adaptor protein downstream of TNFR1 signaling, was reported to be sequestered in SGs following stress, resulting in reduced NF-κB signaling, suggesting stress responses can regulate signaling pathways necessary for TNFR1-dependent necroptosis induction (Kim et al, 2005). The ISR may also modulate the post-translational state of key necroptosis signaling regulators via PERK activity, which was required for thapsigargin stress-mediated inhibition of necroptosis. Different cell types and ISR inducers, which have or induce compositionally different SGs, may also have distinct mechanisms for regulating the response to stressors and the activators of programmed cell death (Aulas et al, 2017; Markmiller et al, 2018; Youn et al, 2018). These differences in cell types may also explain phenotypic differences in necroptosis observed across different cell types and cell lines, as we also observed.

The contribution of necroptosis, the ISR, and SGs to various diseases is still poorly understood. Necroptosis contributes to lethal developmental defects in mice lacking FADD, CASP8, and RIPK1, but mice lacking only RIPK3 or MLKL are viable, suggesting necroptosis plays an important role largely in cases where apoptosis is dysregulated (Dannappel et al, 2014; Dillon et al, 2014; Newton et al, 2016). In humans, MLKL mutations have recently been linked to autoinflammatory diseases and neurodegeneration (Faergeman et al, 2020; Hildebrand et al, 2020). Microbes which interfere with inflammatory signaling and apoptotic pathways have also been shown to induce necroptosis, suggesting necroptosis plays an important role in host protection (Kitur et al, 2016; Yu et al, 2018; Zhang et al, 2020). Similarly, pathogens often inhibit or hijack the ISR to facilitate viral replication or alter inflammatory signaling pathways (Rodrigues et al, 2018; Zhang et al, 2019). The crosstalk between the ISR and necroptosis is likely to have important implications for pathogen control. SGs are also implicated in neurological disorders such as amyotrophic lateral sclerosis, frontal temporal lobar dementia, and fragile X syndrome (Mahboubi & Stochaj, 2017; Wolozin & Ivanov, 2019; Wang et al, 2020), and they can regulate cancer cell growth and responses to chemotherapeutic drugs, suggesting a context- and cell-dependent role for SGs in regulating cancer cell death (Anderson et al, 2015; Zhan et al, 2020). In our study, we identified a significant role for the ISR in negatively regulating necroptosis signaling pathways, which improves our understanding of the mechanisms by which stress responses, SGs, and programmed cell death may broadly be targeted therapeutically.

## Materials and Methods

### Mice

Wildtype (C57BL/6J), *Ripk1*^K45A/K45A^ (indicated as *Ripk1*^KD/KD^ [kinase-dead]) (Berger et al, 2014), *Ripk3*^−/−^ (Newton et al, 2004), *Mlkl*^−/−^ (Murphy et al, 2013), *Lyz2*^cre^*Map3k7*^fl/fl^ (indicated as *Tak1*^fl/fl^) conditional knockout (generated by crossing *B6.129P2-Lyz2*^*tm1(cre)Ifo*^*/J* [The Jackson Laboratory] and *Tak1*^*fl/fl*^) (Xie et al, 2006), and *Lyz2*^cre^*Ddx3x*^fl/fl^ (generated by crossing *B6.129P2-Lyz2*^*tm1(cre)Ifo*^*/J* [The Jackson Laboratory] and *Ddx3x*^fl/fl^) (Samir et al, 2019) mice were maintained on the B6 background. Male and female mice were used in this study at 6–10 wk of age. Mice were bred at St. Jude Children’s Research Hospital, and studies were conducted under protocols approved by St. Jude Children’s Research Hospital Committee on the Use and Care of Animals.

### BMDM culture

Primary BMDMs were grown for 6 d in IMDM (12440-053; Thermo Fisher Scientific) supplemented with 10% FBS (S1620; BioWest), 30% L929-conditioned media, 1× nonessential amino acids (11140050; Gibco), and 1× penicillin-streptomycin (15070063; Thermo Fisher Scientific) (Tweedell et al, 2020). BMDMs were seeded in DMEM (11995-073; Thermo Fisher Scientific) containing 10% FBS and 1× penicillin-streptomycin at a concentration of 1 × 10^6^ cells (12-well plates, for immunoblot analysis) or 5 × 10^5^ cells (24-well plates, for IncuCyte cell death analysis) and incubated overnight. Cells were then washed, cultured, and stimulated in DMEM with 10% FBS.

### MEF culture

MEFs were derived by minimal passage of cells in DMEM containing 10% FBS and 1× penicillin-streptomycin. MEFs were seeded overnight at 1 × 10^5^ cells in a 24-well plate before stimulation.

### Primary BMDM and MEF cell stimulations

Cells were pre-stressed with SG-inducing agents: thapsigargin (2 μg/ml, [10522; Cayman Chemical]); brefeldin A (3 μg/ml [00-4505-51; Thermo Fisher Scientific]); tunicamycin (20 μg/ml [3516; Tocris]); MG132 (25 μM [474790; Calbiochem]); sodium (meta) arsenite (50 μM [S7400; Sigma-Aldrich]); rocaglamide A (250 nM [Hy-19356; MedChemExpress]) 1 h or 30 min (for arsenite) before addition of TNF (25 ng/ml) unless otherwise specified. To induce necroptosis, cells were pretreated where indicated with zVAD (50 μM) 1 h before TNF, LPS (100 ng/ml [tlrl-smlps; InvivoGen]), poly I:C (5 μg/ml [tlrl-pic; InvivoGen]), or TAK1 inhibitor (5Z-7-oxozeaenol [0.1 μM]; Cayman Chemical) treatment. In SG disruption experiments, anisomycin (25 μg/ml) or cycloheximide (25 μg/ml) were added 15 min before TNF, where indicated. Pyroptosis was induced by priming cells for 4 h with LPS (100 ng/ml) followed by addition of nigericin (20 μM [11437; Cayman Chemical]).

### siRNA knockdown of *Perk*

The siGENOME siRNA SMARTpool containing four siRNAs targeting *Perk* (Horizon; M-044901-01-005) was used. A total of 5 nmol was dissolved in nuclease-free water to a concentration of 50 μM, and 0.5 μl siRNA was added to 1 × 10^6^ BMDMs. Electroporation was performed using the Neon transfection system (Invitrogen), with parameters −1,500 V, 1 pulse and 20-ms width. Mock transfection was performed as described but without the addition of siRNA. After electroporation, BMDMs were immediately transferred into 12-well plates with a seeding density of 1 × 10^6^ cells per well. BMDMs were pretreated with thapsigargin and zVAD followed by TNF to induce necroptosis as above 48 h after transfection.

### Microscopy

To image and quantify cell death over time, images were automatically collected using an IncuCyte S3 (Essen Biosciences). To quantify cell death, cells were stimulated in media containing 25 nM Sytox Green (S7020; Thermo Fisher Scientific), and Sytox^+^ nuclei were quantified by automated analysis using the Basic Analyzer module provided with the IncuCyte software (v2018C). Three images were collected for each replicate well (n = 3) in each experiment and Sytox^+^ nuclei counts were exported as Object Count (Sytox^+^ nuclei) per well values, which extrapolates the total object count based on the count per image, area of image acquisition, and the total area of the well. For confocal microscopy, cells were seeded in chamber slides (80055 or 80826, Ibidi). BMDMs were stimulated, then fixed in 4% PFA, permeabilized with 0.1% Triton X-100, blocked in 5% BSA/PBS-T (0.1% Tween-20), and stained with indicated primary antibodies for G3BP1 (66486-1-Ig; Proteintech, 1:250) or DDX3X (A300-474A; Bethyl Laboratories, 1:250) overnight at 4°C in blocking solution. Cells were then washed with PBS-T, incubated with appropriate secondary antibodies conjugated with fluorophores (A-11001, A-11004, A-11008, or A-11011; Thermo Fisher Scientific, 1:250) for 2 h at room temperature, washed, and imaged on a Nikon C2 confocal microscope. SG signal intensity analysis was determined in Imaris 9.3 (Oxford Instruments). SG objects were identified by generating a colocalization channel for G3BP1 (signal threshold 500-4,095)/DDX3X (signal threshold (2,000-4,095)) followed by SG object identification [Surfaces menu] to obtain object signal intensities [IntensitySum] values for all objects (threshold 100-4,095) normalized to media control.

### Immunoblotting analysis

For signaling blots, supernatant was removed, and cells were lysed in RIPA buffer containing protease and phosphatase inhibitors plus 4× Laemmli sample buffer. Proteins were separated via SDS–PAGE with 8–12% polyacrylamide gels, transferred to PVDF membranes (IPVH00010; Millipore), and blocked with 5% nonfat dry milk. Primary antibodies against phospho-MLKL (Ser345) (37333; Cell Signaling Technologies [CST], 1:1,000), MLKL (37705; CST, 1:1,000), phospho-RIPK3 (Thr231/Ser232) (91702; CST, 1:1,000), RIPK3 (2283; ProSci, 1:1,000), phospho-eIF2α (Ser51) (3398; CST, 1:1,000), eIF2α (9722; CST, 1:1,000), RIPK1 (3493; CST, 1:1,000), phospho-RIPK1 (Ser166) (53286; CST, 1:1,000), phospho-RIPK1 (Ser321) (38662; CST, 1:1,000), DDX3X (A300-474A; Bethyl Laboratories, 1:1,000), PERK (3192; CST, 1:1,000), or β-actin (8457; CST, 1:1,000) were incubated overnight at 4°C followed by appropriate secondary antibodies conjugated with HRP incubated for 1 h at room temperature (Jackson ImmunoResearch). Membranes were visualized using Luminata Forte Chemiluminescence substrate (WBLUF0500; Millipore) or SuperSignal West Femto substrate (34096; Thermo Fisher Scientific) on a Bio-Rad ChemiDoc.

### Quantification and statistical analysis

GraphPad Prism 6.0 or Imaris 9.3 software were used for data analysis. Data are shown as mean ± SEM. Statistical significance was determined by *t* test for two groups or one-way ANOVA for three or more groups and two-way ANOVA for comparison between multiple groups. The specific statistical testing for each experiment is indicated in the figure legends.

## Supplementary Material

Reviewer comments
